# Identification of a Novel Di-D-Fructofuranose 1,2’:2,3’ Dianhydride (DFA III) Hydrolysis Enzyme from *Arthrobacter aurescens* SK8.001

**DOI:** 10.1371/journal.pone.0142640

**Published:** 2015-11-10

**Authors:** Shuhuai Yu, Xiao Wang, Tao Zhang, Timo Stressler, Lutz Fischer, Bo Jiang, Wanmeng Mu

**Affiliations:** 1 State Key Laboratory of Food Science and Technology, Jiangnan University, Wuxi, 214122, Jiangsu, People’s Republic of China; 2 Synergetic Innovation Center of Food Safety and Nutrition, Jiangnan University, Wuxi, 214122, Jiangsu, People’s Republic of China; 3 University of Hohenheim, Institute of Food Science and Biotechnology, Department of Biotechnology and Enzyme Science, Garbenstr. 25, 70599, Stuttgart, Germany; University of Edinburgh, UNITED KINGDOM

## Abstract

Previously, a di-D-fructofuranose 1,2’:2,3’ dianhydride (DFA III)-producing strain, *Arthrobacter aurescens* SK8.001, was isolated from soil, and the gene cloning and characterization of the DFA III-forming enzyme was studied. In this study, a DFA III hydrolysis enzyme (DFA IIIase)-encoding gene was obtained from the same strain, and the DFA IIIase gene was cloned and expressed in *Escherichia coli*. The SDS-PAGE and gel filtration results indicated that the purified enzyme was a homotrimer holoenzyme of 145 kDa composed of subunits of 49 kDa. The enzyme displayed the highest catalytic activity for DFA III at pH 5.5 and 55°C, with specific activity of 232 U mg^-1^. *K*_m_ and *V*_max_ for DFA III were 30.7 ± 4.3 mM and 1.2 ± 0.1 mM min^-1^, respectively. Interestingly, DFA III-forming enzymes and DFA IIIases are highly homologous in amino acid sequence. The molecular modeling and docking of DFA IIIase were first studied, using DFA III-forming enzyme from *Bacillus* sp. snu-7 as a template. It was suggested that *A*. *aurescens* DFA IIIase shared a similar three-dimensional structure with the reported DFA III-forming enzyme from *Bacillus* sp. snu-7. Furthermore, their catalytic sites may occupy the same position on the proteins. Based on molecular docking analysis and site-directed mutagenesis, it was shown that D207 and E218 were two potential critical residues for the catalysis of *A*. *aurescens* DFA IIIase.

## Introduction

In general, to grow in nature or in laboratory, the heterotrophic bacteria require carbohydrates as an energy source for cell growth. They secrete extracellular polysaccharide hydrolase to degrade polysaccharides into low-molecular-weight carbohydrates, especially monosaccharides, and further conduct the intracellular catabolism of the carbohydrate hydrolysates to obtain energy for growth. In nature, many polysaccharides can be used as an energy source, such as xylan [1], starch [2], mannan [3], and inulin [4].

**Fig 1:**
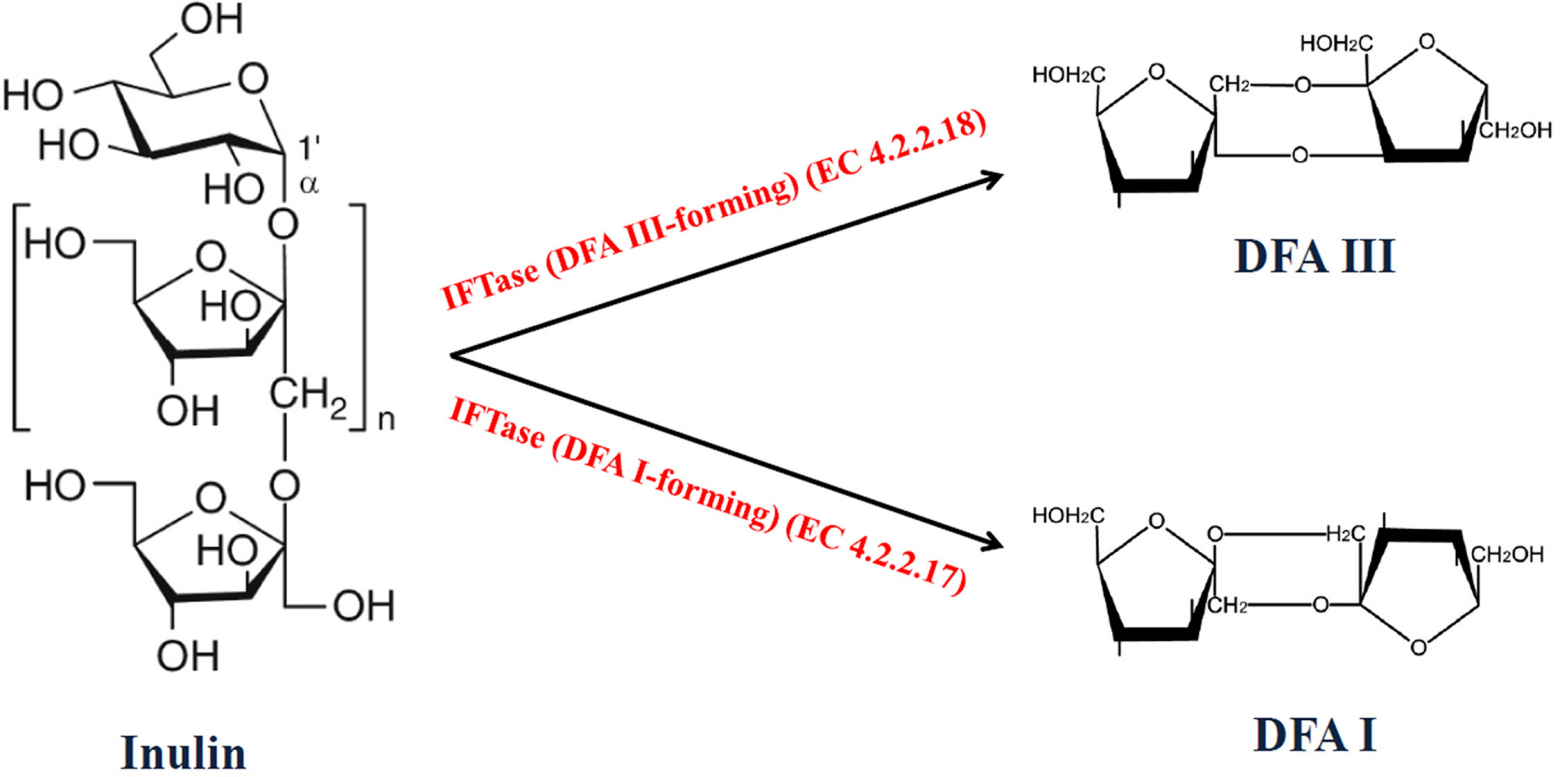
Enzymatic production of DFAs from inulin by IFTases.

Inulin, a type of fructan widely existing in plants, is a type of polysaccharide composed mainly of fructose units terminated by glucose residue (Fig 1). A wide range of microorganisms can biologically utilized inulin. Inulin hydrolysis can be catalyzed by microbial inulinases, in which exoinulinase (EC 3.2.1.80) hydrolyzes the terminal, non-reducing β-D-fructofuranose residues from inulin chain producing monosaccharide fructose [5]. Endoinulinase (EC 3.2.1.7) reduces the long chain of inulin into shorter fructooligosaccharides [6]. In recent years, a new type of inulinase named inulin fructotransferase (IFTase) was found, which catalyzes the inulin hydrolysis to difructose dianhydrides (DFA) [7]. Two types of DFAs have been produced from inulin, including DFA III (α-D-fructofuranose-β-D-fructofuranose 2’,1:2,3’-dianhydride) and DFA I (α-D-fructofuranose-β-D-fructofuranose 2’,1:2,1’-dianhydride), by IFTase (DFA III-forming) (EC 4.2.2.18) and IFTase (DFA I-forming) (EC 4.2.2.17), respectively (Fig 1) [8, 9]. According to the CAZy database information, both enzymes are classified as members of glycoside hydrolase family 91. Biological production of DFAs by IFTases has attracted much attention [10–13] because they show great potential in food and beverage industries, due to their low calorie properties [14] and beneficial effects such as displaying prebiotic activity [15–17] and improving the absorption of minerals [18–20], flavonoids [21], and immunoglobulin G [22].

So far, approximately 20 microbial strains have been isolated that can produce DFAs from inulin, and most of them are *Arthrobacter* species [8]. Many experimental results show that inulin is an important inducer for IFTase production during the fermentation of DFA-producing bacteria [23–25]. In addition, some IFTase-producing strains may grow well by using inulin as a sole carbon source, accompanied by a significant increase of the IFTase expression level [23, 26]. Therefore, it has been suggested that IFTase participates in the inulin metabolism by converting inulin to DFA [8]. However, very few studies focus on how these strains further utilize DFA as energy source. Previously, researchers identified the DFA III hydrolysis enzymes (DFA IIIase, EC 3.2.1.-, glycoside hydrolase family 91) from *Arthrobacter ureafaciens* and *Arthrobacter* sp. H65-7 and proposed that DFA IIIase hydrolyzes DFA III to inulobiose, inulobiose is further hydrolyzed into two fructose molecules by β-fructofuranosidase, and then fructose becomes the energy source for cell growth [26–28]. Thus far, gene cloning of DFA IIIase and characterization of the recombinant DFA IIIase have been only investigated by Saito et al. from *Arthrobacter* sp. H65-7 [29]. The DFA IIIase from *Arthrobacter* sp. H65-7 shows a relatively close relationship with IFTase (DFA I-forming) and IFTase (DFA III-forming) based on phylogenetic tree analysis [29]; in addition, it shows 44–47% of amino acid identity with all the reported IFTases [8].

In our previous studies, an IFTase (DFA III-forming)-producing microorganism, *Arthrobacter aurescens* SK8.001, was isolated using inulin as a sole carbon and energy source [30]. Specially, IFTase (DFA III-forming) was significantly induced when inulin was used as sole carbon source; Moreover, the gene encoding IFTase (DFA III-forming) from *A*. *aurescens* SK8.001 was sequenced (GenBank accession No. HM138085) and the extracellular overexpression was further studied in *Escherichia coli* [31]. When inulin was used as a sole carbon source for *A*. *aurescens* SK8.001 growth, high amounts of DFA III but no fructose were detected in the extracellular culture broth [23]; therefore, it was suggested that DFA III was first assimilated into the cells of *A*. *aurescens* and was probably converted by DFA IIIase to inulobiose for further catabolism. Regarding this probable metabolic pathway of DFA III, it is proposed that *A*. *aurescens* SK8.001 might have a DFA IIIase-encoding gene similar to that of *Arthrobacter* sp. H65-7. In the current article, the DFA IIIase-encoding gene from *A*. *aurescens* SK8.001 was obtained and sequenced, and the recombinant enzyme was identified and characterized, in addition, the potential residues of active site of DFA IIIase were first proposed based on homology modeling and molecular docking accompanied with site-directed mutagenesis. The DFA IIIase from *A*. *aurescens* SK 8.001 was abbreviated as *Aa*DFA IIIase in this study.

## Materials and Methods

### Chemicals and reagents

The resin for protein purification, the Chelating Sepharose Fast Flow, was obtained from GE (Uppsala, Sweden). The standard of DFA III was purchased from Wako Pure Chemical Industries (Osaka, Japan). Electrophoresis reagents were purchased from Bio-Rad (Hercules, CA, USA). Isopropyl-β-D-1-thiogalactopyranoside (IPTG) and all chemicals used for enzyme assays and characterization were at least of analytical grade obtained from Sigma (St. Louis, MO, USA) or Sinopharm Chemical Reagent (Shanghai, China).

### Plasmids, bacterial strains, and culture conditions

The pET-22b(+) expression vector was obtained from Novagen (Darmstadt, Germany). *A*. *aurescens* SK 8.001 was isolated by our laboratory previously from soil and kept in China Center for Type Culture Collection (CCTCC) under the accession number of M 207185. The strain was grown at 30°C and 200 rpm for 12 h in flask containing culture medium (peptone 5 g L^-1^, NaCl 5 g L^-1^, and yeast extract 3 g L^-1^, pH 7.0). *E*. *coli* strains DH5α and BL21(DE3) were obtained from Sangon Biological Engineering Technology and Services (Shanghai, China), which were used as the host strains for DNA cloning and expression, respectively. The recombinant *E*. *coli* strains were routinely grown in Luria-Bertani (LB) medium with ampicillin (100 μg mL^-1^) in a rotary shaker at 37°C and 200 rpm.

### Gene cloning and expression

The genomic DNA was extracted from harvested cells of *A*. *aurescens* SK8.001 using the Genomic DNA Isolation Kit (Sangon, Shanghai, China) according to the manufacturer’s protocol. The complete genome sequence of *A*. *aurescens* TC1 has been released in GenBank (NCBI accession number: CP000474.1), and revealed that a putative IFTase gene was included in the genome (gene locus_tag: AAur_0696; protein ID: ABM07149.1). The sequence of the oligonucleotide primers used for gene cloning was based on the putative gene. Forward (5’-CGCCATATGGAAATTGACGAGACGCTT-3’) and reverse (5’-TATACTCGAGTCGAGGAATACCCGCGCC) primers were designed to introduce the *Nde*I and *Xho*I restriction sites (underlined). The polymerase chain reaction (PCR) was performed by Taq Plus DNA polymerase for 35 cycles consisting of 94°C for 30 s, 57°C for 1 min, and 72°C for 1 min, followed by a final extension step at 72°C for 10 min. The amplified DNA fragment (1.4 kb) was extracted using the SanPrep column DNA gel extraction kit and was sequenced by Sangon Biological Engineering Technology and Service (Shanghai, China). The target fragment was fused with a 6× histidine-tag at the C-terminal, digested using *Nde*I and *Xho*I, and then was inserted into the same restriction sites of pET-22b(+) plasmid.

The reconstructed plasmid was transformed into *E*. *coli* BL21(DE3) for overexpression of the recombinant DFA IIIase. For protein expression, the recombinant *E*. *coli* BL21(DE3) was cultivated in 500 mL LB medium supplemented with 100 μg mL^-1^ ampicillin in a rotary shaker at 37°C and 200 rpm. When the absorbance (600 nm) of the bacterial culture reached 0.6, IPTG was added at a final concentration of 0.5 mM and the cells were grown at 28°C for an additional 6 h.

### Purification of the recombinant DFA IIIase

The cells were collected by centrifugation at 10,000 × *g* (4°C, 10 min), washed with phosphate buffer (pH 7.0, 50 mM), and disrupted by sonication at 4°C for 6 min (pulsations of 3 s, amplify 90) using a Vibra-Cell^TM^ 72405 Sonicator (Sonics, Newtown, CT, USA). The lysates were centrifuged at 20,000 × *g* (4°C, 30 min) to remove the cell debris. As fused with a 6×histidine tag, the expressed enzyme was allowed to be purified by nickel-affinity chromatography. The supernatant was loaded onto a Ni^2+^-chelating Sepharose Fast Flow column (GE Healthcare, Uppsala, Sweden) equilibrated with a binding buffer (50 mM sodium phosphate buffer, 500 mM NaCl, pH 7.5). The unbound proteins were eluted from the column using a washing buffer (50 mM sodium phosphate buffer, 500 mM NaCl, 50 mM imidazole, pH 7.5), and then the recombinant enzyme was eluted by using a elution buffer (50 mM sodium phosphate buffer, 500 mM NaCl, 500 mM imidazole, pH 7.5). To remove imidazole, the collected fractions were dialyzed against sample buffer (50 mM citrate buffer, pH 5.5). After dialysis, the resulting solution was used as the purified enzyme for further studies.

### Protein concentration, electrophoresis, and gel filtration chromatography

According to the method of Bradford [32], the protein concentration was determined using bovine serum albumin as a standard. The molecular mass and purity of the purified *A*. *aurescens* DFA IIIase were examined by denaturing discontinuous sodium dodecyl sulfate-polyacrylamide gel electrophoresis (SDS-PAGE) on a 5% stacking gel and a 12% separating gel as described by Laemmli [33]. Gels were stained with Coomassie Brilliant Blue R250 and destained with an aqueous mixture of 10% (V/V) methanol/10% (V/V) acetic acid at room temperature. Native molecular weight was estimated by gel filtration using high-performance liquid chromatography (HPLC) at a flow rate of 1 mL min^-1^ (column: TSK G2000SWxl, Tosoh Bioscience LLC, Tokyo, Japan; mobile phase: 0.1 M phosphate buffer, pH 6.7, containing 0.05% (W/V) NaN_3_ and 0.1 M Na_2_SO_4_; detection: UV at 280 nm).

### Enzyme assay

The enzyme activity was measured by the determination of the amount of produced inulobiose from DFA III. The enzyme reactions were carried out at 55°C for 10 min in a 1 mL reaction mixture containing DFA III (10 g L^-1^), citrate buffer (50 mM, pH 5.5), and 100 nM enzyme, and stopped by boiling for 10 min. The mixture was centrifuged for 30 min at 20,000 × *g*, and the supernatant was filtered through a 0.22 μm membrane filter into a sample vial for HPLC analysis. The carbohydrates were determined by HPLC (Agilent 1200 system, Agilent technologies, Santa Clara, CA, USA) equipped with a refractive index detector and an Asahipak NH2P-5004E column (4.6 mm × 250 mm, Shodex, Tokyo, Japan; column temperature: 25°C; mobile phase: 65% (V/V) acetonitrile; flow rate: 1 mL min^-1^). One unit of DFAIIIase was defined as the amount of enzyme that produces 1 μmol inulobiose per min at pH 5.5 and 55°C.

### Determination of the reaction product from DFA III by the purified DFA IIIase

For preparation of reaction products, the purified DFA IIIase solution (100 nM) and 100 g L^-1^ (W/V) DFA III were mixed in a 100 mL enzyme reactor and maintained at 55°C and pH 5.5 for 2 h. The reaction temperature was controlled with circulating water bath and the mixture was heated at 100°C for 10 min to terminate the enzyme reaction. Thereafter, the mixture was centrifuged at 20,000 × *g* (4°C, 30 min) and supernatant was filtrated through a 0.22 μm membrane filter. The filtrate was then applied to a preparative column (10.0 × 300 mm, Sepax, Newark, Delaware, USA) with HPLC (Agilent 1200 system) equipped with a refractive index detector (temperature: 80°C, mobile phase: 2.5 mM trifluoroacetic acid; flow rate: 0.5 mL min^-1^; injection volume: 200 μL). The fraction of product was collected and pooled in a 50 mL centrifuge tube and lyophilized with a vacuum freezing dryer. The dried product was dissolved in deuteroxide (D_2_O) and analyzed by ^13^C-NMR system 300 spectrometer (Varian, Palo Alto, CA, USA) using 3-trimethylsilyl-1-pro-panesulfonic acid sodium salt (DSS) as a standard.

In addition, the ultra-performance liquid chromatography and electrospray ionization quadrupole time-of-flight mass spectrometry (UPLC/ESI-QTOF-MS, SYNAPT™ High Definition Mass Spectrometry™ system; Waters, Milford, MA, USA) was used for the product analysis. The system equipped with a TSK-gel Amide-80 column (4.6 mm id × 25 cm, 5 μm, 80 Å, Tosoh, Tokyo, Japan) was operated in the negative ion mode with a negative electrospray ionization source (ESI^-^), and the cone voltage, capillary voltage, source temperature, and mass range were set as 30 V, 3.0 kV, 100°C, and 50–1000 m/z, respectively.

### Effect of pH and temperature on the enzyme activity

The optimum pH was determined using two buffer systems with different pH values at 55°C for 10 min, including sodium citrate buffer (50 mM, pH 4.0–5.5) and sodium phosphate buffer (50 mM, pH 6.0–8.0). The effect of temperature on the activity of the *Aa*DFA IIIase was analyzed by assaying the enzyme samples over the range of 30–60°C at pH 5.5 for 10 min.

### Determination of kinetic parameters

To determine kinetic parameters of the *Aa*DFA IIIase, various concentrations of DFA III (2.0–50 mM) in 50 mM of citrate buffer (pH 5.5) was incubated with the enzyme at 55°C for 10 min, and the reactions were stopped by heating in boiling water for 10 min. The inulobiose produced was measured by HPLC assay method. The Michaelis-Menten constant (*K*_m_) and the maximum rate (*V*_max_) values were estimated by a nonlinear regression method.

### Molecular modeling and docking

To obtain a template of molecular modeling, the amino sequence of *Aa*DFA IIIase was submitted to SWISS-MODEL protein-modeling server (http://www.expasy.ch/swissmod/SWISS-MODEL.html) with Automated Mode [34–37], and the crystallographic structure of *Bs*IFTase from *Bacillus* sp. snu-7 (PDB ID: 2INU) was selected as the template of homology modeling for *Aa*DFA IIIase and its mutants. Stereochemical quality of the modeled structures were assessed by analyzing Ramachandran map using WinCoot, the compatibility of an atomic model (3D) with its own amino acid sequence (1D) was determined by VERIFY-3D (a structure evaluation server) [38] through ASVES server (http://services.mbi.ucla.edu/SAVES/).

Molecular docking of DFA III to *Aa*DFA IIIase and mutants models was carried out with the Autodock4.2 software package [39]. The structure of ligand DFA III was obtained from PubChem (http://pubchem.ncbi.nlm.nih.gov/) (CID of DFA III: 196181), and this structure was transformed to pdb format with the GlycoBioChem PRODRG2 Server [40]. Proteins were added with polar hydrogen atoms using the Hydrogen module in AutoDock Tools (ADT), and all the torsion angles in the DFA III were set free to perform flexible docking. The empirical free energy function and Lamarckian genetic algorithm (LGA) were used for docking with the following settings: a population size of 150 dockings and 5 million energy evaluations, a maximum number of 27,000 generations, a mutation rate of 0.02, a crossover rate of 0.80 and an elitism value (number of top individuals that automatically survive) of 1. Fifty independent docking runs were carried out for each ligand. Results were clustered according to the root-mean-square deviation (RMSD) criterion. The best docked conformations were selected as the initial conformations to evaluate the molecular docking between the enzyme and the ligands. The figures were prepared using Discovery Studio Visuallizer 4.0 (Accelrys, San Diego, USA)/Pymol.

### Mutagenesis

Site-directed mutagenesis of the *Aa*DFA IIIase gene of *A*. *aurescens* SK8.001 was implemented by one-step PCR method, the aforementioned recombinant plasmid harboring the wild-type *Aa*DFA IIIase gene was used as template and a pair of synthetic complementary oligonucleotides were used as primers. Oligonucleotides shown in Table 1 were synthetized by Shanghai Generay Biotech Co., Ltd (Shanghai, China) and the underlined sequences indicated mutated codons. The amplified PCR products were digested with *Dpn*I restriction enzyme and then transformed into *E*. *coli* DH5α host cells. The nucleotide sequences of mutants were verified by DNA sequencing. For expression of mutant enzymes, the plasmids containing the various mutant gene of *Aa*DFA IIIase were introduced into *E*.*coli* BL21(DE3) host cells. The purification and enzyme assay of these mutants were performed as those of the wild-type *Aa*DFA IIIase.

**Table 1:** Primers for site-directed mutagenesis. The underlined sequences represented mutated codons.

Primers	Oligonucleotides
For D207A	Forward: ACCATCTGCAAGGCAGCTGCCCTGTCCGTC
	Reverse: TGCCTTGCAGATGGTGAGGCGCAAGAGTTC
For D207N	Forward: ACCATCTGCAAGGCAAACGCCCTGTCCGTC
	Reverse: TGCCTTGCAGATGGTGAGGCGCAAGAGTTC
For E218A	Forward: GACAACTTCATTGCTGCTTGCGGATCGACG
	Reverse: AGCAATGAAGTTGTCGTGGACGGACAGGGC
For E218N	Forward: GACAACTTCATTGCTAACTGCGGATCGACG
	Reverse: AGCAATGAAGTTGTCGTGGACGGACAGGGC

## Results and Discussion

### Gene cloning and sequence analysis

Through searching the genome information from the GenBank, it was found that a same species bacterium, *A*. *aurescens* TC1, was shown in the database with known complete genome sequence information (NCBI accession No.: CP000474.1) [41]. The genome sequence revealed the presence of a putative gene but unidentified IFTase gene (locus_tag: AAur_0696; protein ID: ABM07149.1). Primers were designed based on the DNA sequence of the AAur_0696 gene, and a hypothetical gene was obtained by PCR amplification from *A*. *aurescens* SK8.001 as template. The nucleotide sequence was submitted to the GenBank database under accession number KR534324. The whole nucleotide sequence is shown in (S1 Fig). The sequence revealed an open reading frame of 1,413 bp, encoding a polypeptide of 470 amino acid residues with a calculated molecular mass of 49,879 Da. The DNA sequence showed 95.57% identity with the AAur_0696, a putative gene but unidentified IFTase gene from *A*. *aurescens* TC1. However, in this article, the encoded protein was identified as a DFA IIIase, not an IFTase; therefore, the gene with locus_tag: AAur_0696 in *A*. *aurescens* TC1 [41] is possibly a putative DFA IIIase rather than an IFTase gene. In addition, the DNA sequence of *A*. *aurescens* SK8.001 DFA IIIase gene showed 79.07% identity with the only identified DFA IIIase gene (GenBank No. AB088205.1) from *Arthrobacter* sp. H65-7 [29].

**Fig 2:**
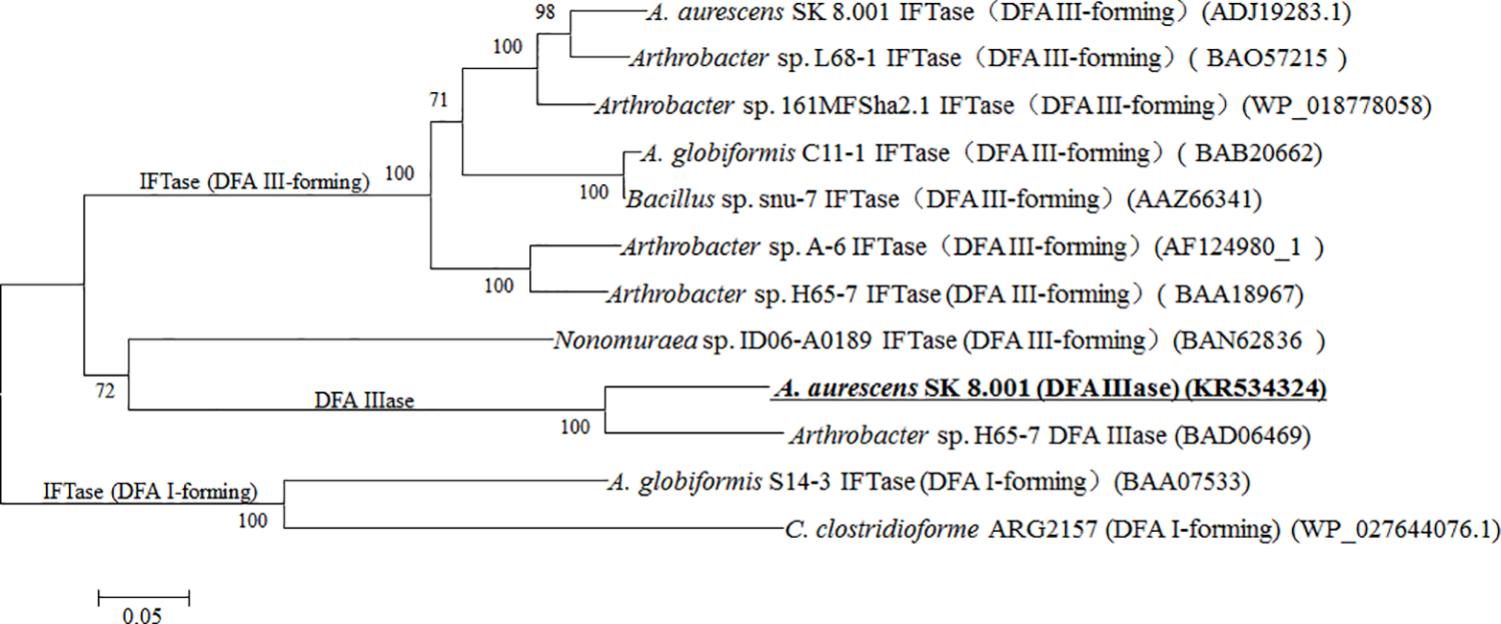
A phylogenetic tree of the reported DFA IIIases and IFTases. The dendrogram was constructed by the neighbor-joining method from the amino acid sequences in Mega5.2 program. Bootstrap values of each branch were obtained as 1,000 by 1,000-repeated bootstrapping. The scale bar indicated the amino acid substitution per site. GenBank accession numbers of the enzymes were showed in the parentheses after each enzyme name.

As shown in Table 2, the deduced amino acid sequence of *Aa*DFA IIIase shared 81.35% identity with the DFA IIIase from *Arthrobacter* sp. H65-7 (GenBank No. BAD06469.1) and 44% to 49% identity with all reported IFTases from various sources. Furthermore, Fig 2 shows a phylogenetic tree of DFA IIIases and all reported IFTases. The dendrogram indicates that DFA IIIases and IFTases from *Arthrobacter* genus form two phylogenetically independent groups and that IFTases are further split into two independent groups (DFA I-forming and DFA III-forming IFTase groups). All these divergences probably indicate that IFTases and DFA IIIases have a relatively close evolutionary relationship but perform different biological functions.

**Table 2:** Identity of amino acid sequences of the reported DFA IIIases and IFTases from various microorganisms.

No.	1	2	3	4	5	6	7	8	9	10	11	12
1	100.00	81.35	41.33	45.04	46.67	44.00	45.25	44.22	44.67	46.79	46.22	48.53
2		100.00	41.07	44.78	46.74	45.17	45.93	44.49	44.49	46.56	46.68	48.75
3			100.00	63.01	44.13	44.39	44.39	44.64	44.39	44.39	43.11	46.17
4				100.00	49.11	48.09	48.60	51.40	51.65	49.62	50.64	50.64
5					100.00	90.00	89.82	80.00	80.44	78.67	79.41	53.51
6						100.00	90.27	78.22	78.89	77.98	78.72	52.83
7							100.00	78.73	79.41	77.75	78.72	53.06
8								100.00	98.89	75.00	76.20	53.51
9									100.00	75.92	77.12	53.74
10										100.00	90.14	53.67
11											100.00	54.00
12												100.00

Nos. 1–2 represented DFA IIIases from *A*. *aurescens* SK 8.001 (GenBank accession No.: KR534324) and *Arthrobacter* sp. H65-7 (BAD06469) [29], respectively; No. 3–4 represented IFTase (DFA I-forming) from *Clortridum clortridioforme* AGR2157 (WP_027644076.1) [47], and *A*. *globiformis* S14-3 IFTase (BAA07533) [48]; Nos. 5–12 represented IFTases (DFA III-forming) from *Arthrobacter* sp. 161MFSha2.1 (WP_018778058) [49], *A*. *aurescens* SK 8.001 (ADJ19283.1) [31], *Arthrobacter* sp. L68-1 (BAO57215) [50], *A*. *globiformis* C11-1 (BAB20662) [51], *Bacillus* sp. snu-7 (AAZ66341) [52], *Arthrobacter* sp. A-6 (AF124980_1) [53], *Arthrobacter* sp. H65-7 (BAA18967) [54], and *Nonomuraea* sp. ID06-A0189 (BAN62836) [55], respectively.

### Purification of the *Aa*DFA IIIase

**Fig 3:**
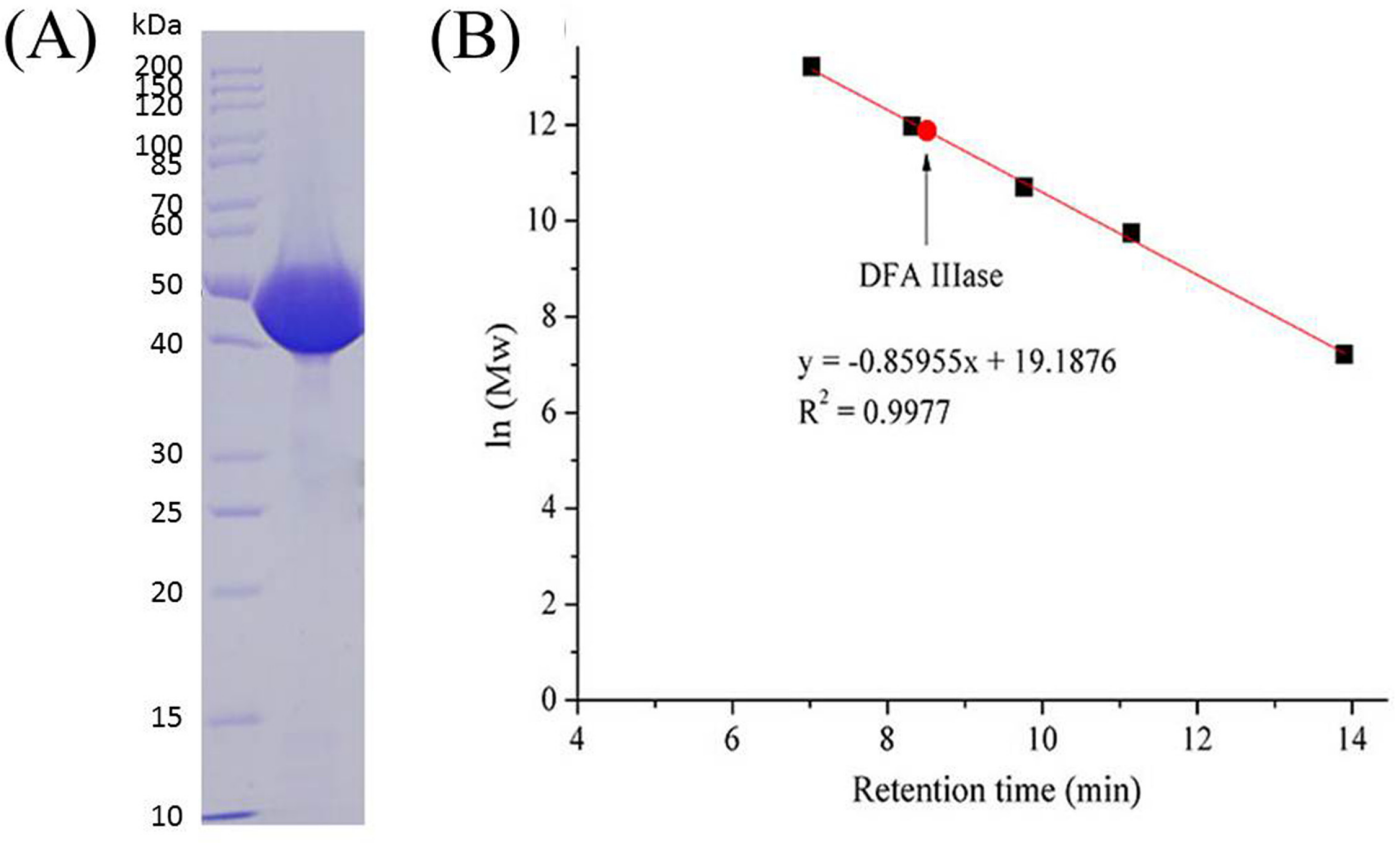
Estimation of the molecular mass of *Aa*DFA IIIase by SDS-PAGE and gel filtration. (A) SDS-PAGE analysis of *Aa*DFA IIIase. The protein and markers were stained with Coomassie Blue. (B) Gel filtration analysis of *Aa*DFA IIIase. The marker proteins include thyroglobulin (bovine, Mw: 670 kDa), γ-globulin (bovine, Mw: 158 kDa), ovalbumin (chicken, Mw: 44 kDa), myoglobin (horse, Mw: 17 kDa), and vitamin B12 (Mw: 1.35 kDa), respectively. The retention times of above corresponding markers are 7.0, 8.3, 9.7, 11.1 and 13.9 min, respectively, and 8.5 min for *Aa*DFA IIIase.

The analysis of the deduced amino acid sequence using SignalP 4.1 Server (http://www.cbs.dtu.dk/services/SignalP/) revealed that there was no signal peptide in *Aa*DFA IIIase. Due to fusing with a 6×histidine-tag (as mentioned in Materials and Methods), the recombinant *Aa*DFA IIIase was allowed to be purified from cell-free extract through a Ni^2+^-chelating affinity chromatography column by one step. Shown in Fig 3A, the enzyme was purified to electrophoretic homogeneity with an estimated molecular mass of 49 kDa, which agreed well with 49,879 Da calculated based on the deduced amino acid sequence. The gel filtration showed that the native purified enzyme had a total molecular mass of approximately 145 kDa (Fig 3B). These results suggested that the *Aa*DFA IIIase is a trimer with three identical subunits. By contrast, the DFA IIIase from *Arthrobacter* sp. H65-7 is a dimer, and its molecular mass, as assayed by SDS-PAGE, was 61 kDa while native total molecular weight as determined by gel filtration was 125 kDa.

Previously, the crystal structure of the IFTase (DFA III-forming) from *Bacillus* sp. snu-7 (*Bs*IFTase) revealed that trimerization is a prerequisite for the catalytic reaction as the active site is located at the monomer-monomer interface. DFA IIIases (EC 3.2.1.-) and IFTases (EC 4.2.2.17, and EC 4.2.2.18) were categorized as glycoside hydrolase (GH) family 91 and had a relatively high sequence identity [42]; therefore, it was suggested that the oligomerization is probably essential for the catalytic reaction of *Aa*DFA IIIase as *Bs*IFTase.

## Identification of Reaction Products

To determine the reaction product, a preparative column was used to separate the product from reaction mixture. There was only one product in the reaction mixture (data not shown), which was analyzed by both NMR and LC-ESI-MS. As shown in the S2 Fig, the purified product exhibited the (M—H)^-^ ion peak of *m/z* 341 Da, indicating that the molecular weight should be 342 Da. In addition, the ^13^C-NMR spectrum (shown in the Table 3) indicated that the chemical shift agreed well with those of reducing and non-reducing terminal fructose of inulobiose. Hence, the product was identified as the inulobiose. Moreover, given the chemical shift of C2 of the reducing terminal fructose, the inulobiose was determined as 1-*O*-*β*-D-fructofuranosyl-D-fructopyranose according to the method described by Sakurai et al. [26]. Therefore, the inulobiose produced by *Aa*DFA IIIase in this work had the same chemical structure as *Arthrobacter* sp. H65-7 DFA IIIase, but was different from *A*. *ureafaciens* DFA IIIase, which was 1-*O*-*β*-D-fructofuranosyl-D-fructofuranose [27]. These results indicated that the recombinant enzyme from *A*. *aurescens* SK8.001 characterized herein should be a DFA III hydrolysis enzyme, DFA IIIase.

**Table 3:** ^13^C-NMR chemical shifts of enzymatic product and standard inulobiose.

Assignment of carbon atom number	Chemical shifts ^*a*^ of ^13^C-NMR resonance of			
	Reducing fructose		Non-reducing terminal	
	Sample	Inulobiose^*b*^	Sample	Inulobiose^*b*^
1	64.70	64.21	62.81	61.00
2	98.81	98.62	101.86	104.39
3	68.49	68.86	75.74	77.83
4	70.57	70.21	75.19	74.97
5	70.02	69.84	81.76	81.94
6	64.24	64.50	62.79	62.73

^*a*^ δ in ppm at 100 MHZ with DSS as a standard.

^*b*^ Reference: [26].

Then, why does the *A*. *aurescens* SK8.001 contain a DFA IIIase-encoding gene? It is suggested that the DFA IIIase is probably involved in the catabolic pathway of inulin in *A*. *aurescens* SK8.001. In our previous studies, *A*. *aurescens* SK8.001 grew very well, accompanied by the biological accumulation of DFA III in broth, when using inulin as the sole carbon source [23]. After DFA III production by the IFTase (DFA III-forming), the strain might need to obtain energy through DFA III hydrolysis by DFA IIIase and further catabolic reactions [8]. So far, there have been very few studies focusing on microbial inulin catabolism via DFA. In 1975, Tanaka et al. reported that the enzymes from autolysate of *A*. *ureafaciens* converted DFA III to fructose with inulobiose as an intermediate [27]. The DFA IIIases from *A*. *ureafaciens* and *Arthrobacter* sp H65-7 were identified to be able to hydrolyze DFA III by DFA IIIase to inulobiose [26, 28]. In addition, Matsuyama et al. found that DFA I and inulobiose could be reversibly converted [43]. For inulin-assimilation though DFA III as intermediate, Saito et al. proposed that inulin is firstly depolymerized to the DFA III by IFTase (DFA III-forming), DFA III is further hydrolyzed to inulobiose by DFA IIIase, and then inulobiose is hydrolyzed to two fructose molecules to enter the tricarboxylic acid (TCA) cycle for generating energy [29]. The enzyme hydrolyzing inulobiose into fructose has not been identified yet, but was proposed to be a type of β-fructofuranosidase [29].

### Effect of pH and temperature on the *Aa*DFA IIIase activity

**Fig 4:**
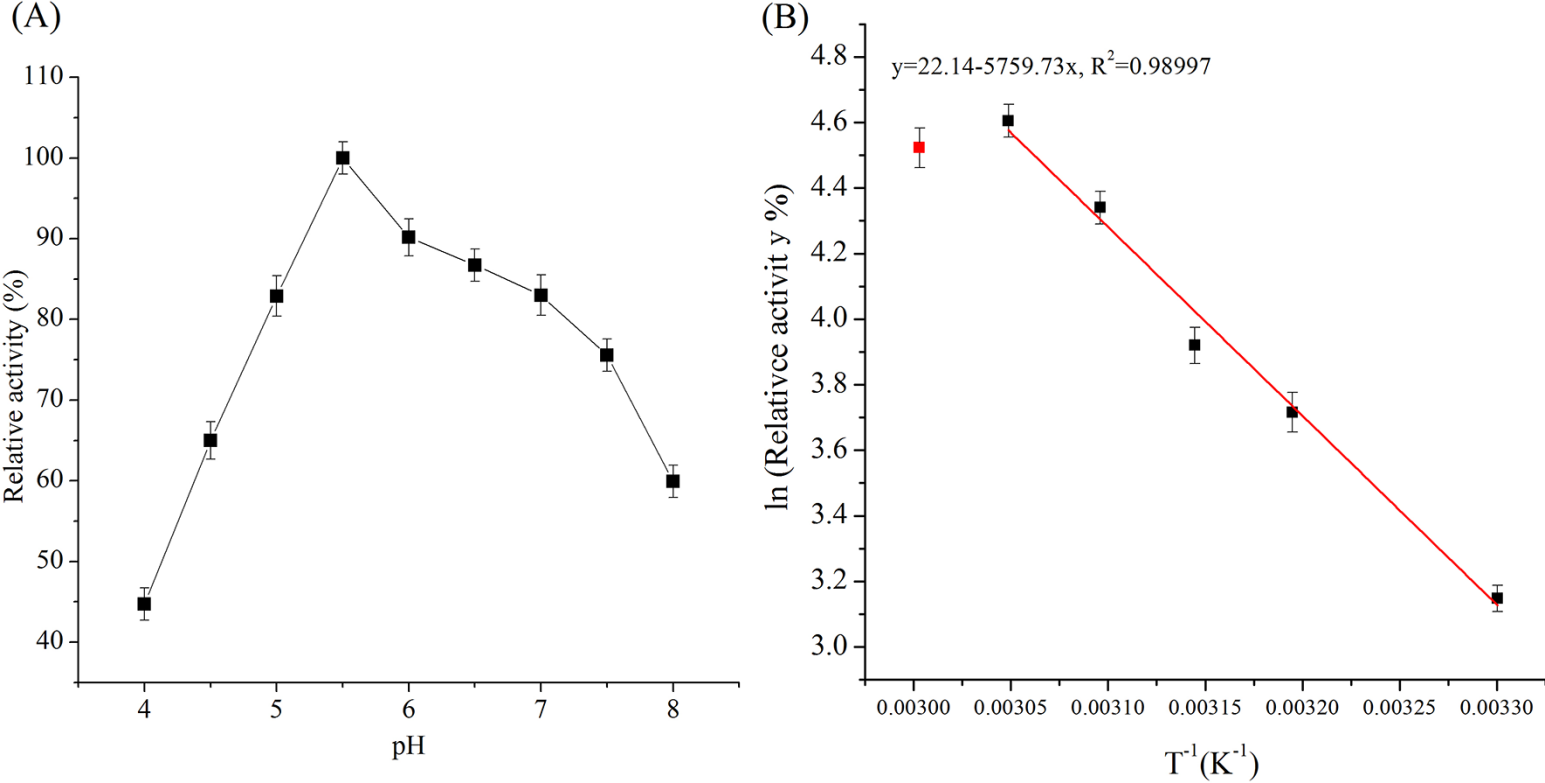
Effect of pH (A) and temperature (B) on the activity of *Aa*DFA IIIase. (A) The relative activity was investigated at 55°C and different pH values. (B) The relative activity was investigated at temperatures varying from 30–60°C at pH 5.5. Data were plotted as ln (relative activity, %) versus T^-1^. Relative activity was expressed as a percentage of the maximal enzyme activity. Values were the means of three replications ± standard deviation.

Shown in Fig 4A, the enzyme exhibited a relatively wide pH spectrum (from 5.0–7.0, approximately above 80% relative activity), with the highest activity at pH 5.5. The optimum pH (pH 6.0) was close to that of DFA IIIase from *Arthrobacter* sp. H65-7 [26]. The enzyme showed the highest activity at 55°C and the activation energy for the hydrolysis reaction calculated from Arrhenius equation was 47.8 kJ mol^-1^ (Fig 4B). The maximum enzymatic activity of 232 ± 15 U/mg was achieved at pH 5.5 and 55°C. By comparison, optimum pH and temperature of the DFA IIIases from *A*. *ureafaciens* ATCC21124 were pH 6.5 and 60°C [28], while the enzyme from *Arthrobacter* sp. H65-7 showed the highest activity at a lower temperature (45°C) [26].

### Enzyme kinetics

The *K*_m_ and *V*_max_ were determined by using nonlinear regression plots in three independent experiments, and the values were 30.7 ± 4.3 mM and 1.2 ± 0.1 mM min^-1^, respectively (S3 Fig). The *K*_m_ of *Aa*DFA IIIase for DFA III was similar to the one from *A*. *ureafaciens* ATCC21124 (31 mM) [28] but higher than the one from *Arthrobacter* sp. H65-7 (12.5 mM) [26].

### Homology building

**Fig 5:**
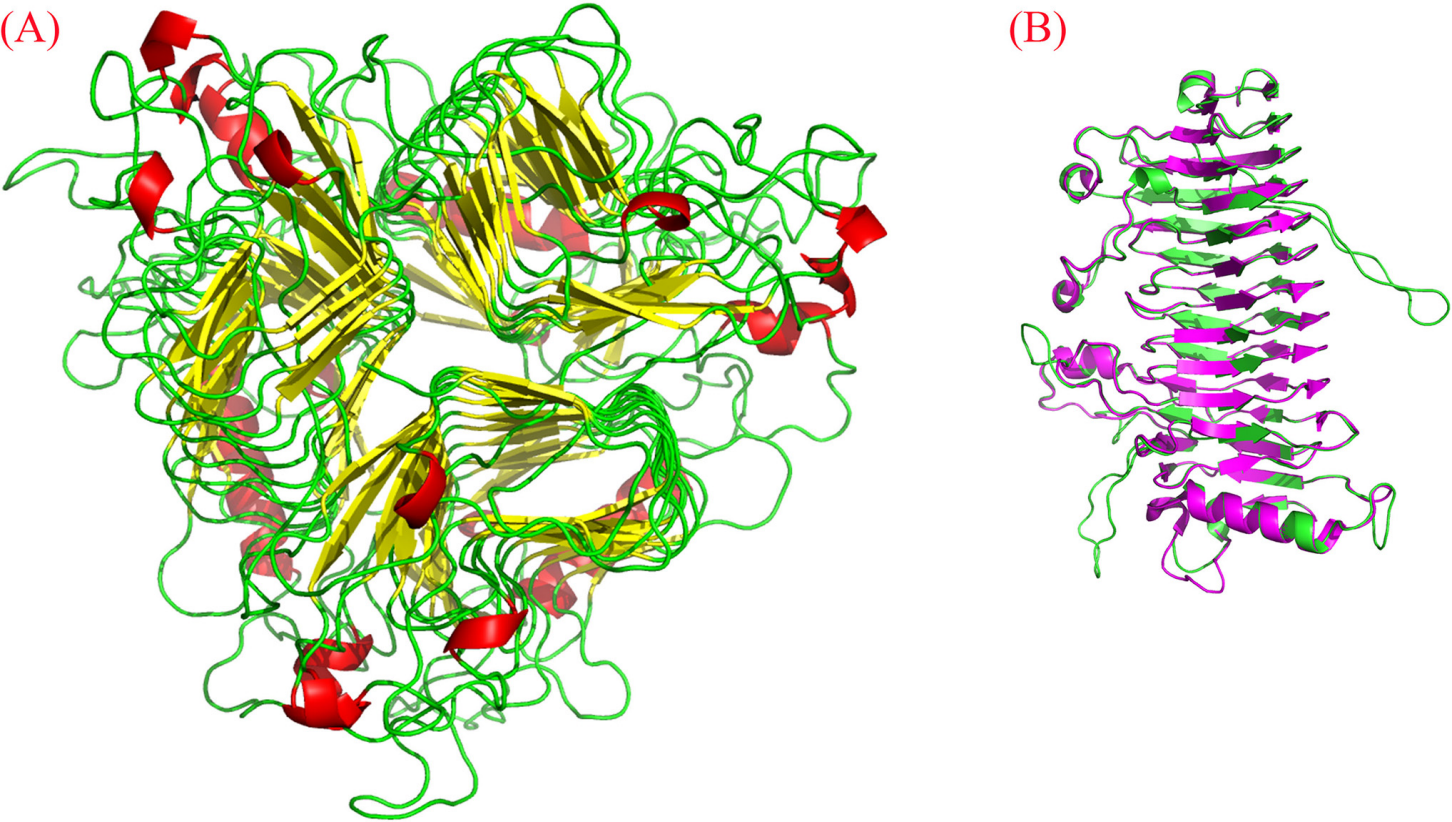
Three-dimensional model of *Aa*DFA IIIase predicted by SWISS-MODEL. (A) A model structure of *Aa*DFA IIIase which shows a typical right-handed parallel *β*-helix. The models of mutants (D207A, D207N, E218A, and E218N) have similar three-dimensional structure with the wild-type enzyme. (B) Superimposition of the monomer of *Aa*DFA IIIase (green) and IFTase from *Bacillus* sp. snu-7 (pink).

So far, the structure of DFA IIIase has not been determined yet. The amino acid sequence of *Aa*DFA IIIase was submitted to SWISS-MODEL with the Automated Mode and compared to proteins with known structure information. The results revealed that *Aa*DFA IIIase had the highest identity (50.88%) with IFTase (DFA III-forming) from *Bacillus* sp. snu-7 (PDB ID: 2INU) but shared less than 30% identity with the others. A model of tertiary structure of *Aa*DFA IIIase was constructed based on the crystallographic structure of *Bacillus* sp. IFTase (as shown in Fig 5A). The generated model was then validated by Ramachandran plot. As shown in S4 Fig, 90.79% of amino acid residues were located in preferred regions, 4.72% of the residues were located in allowed regions, and 4.49% of the residues were located in outlier regions of the Ramachandran plot. VERIFY-3D analysis showed that 83% of the residues had an averaged 3D-1D score ≥ 0.2. The models of various mutants had similar results. These results indicated that the predicted models were acceptable.

**Fig 6:**
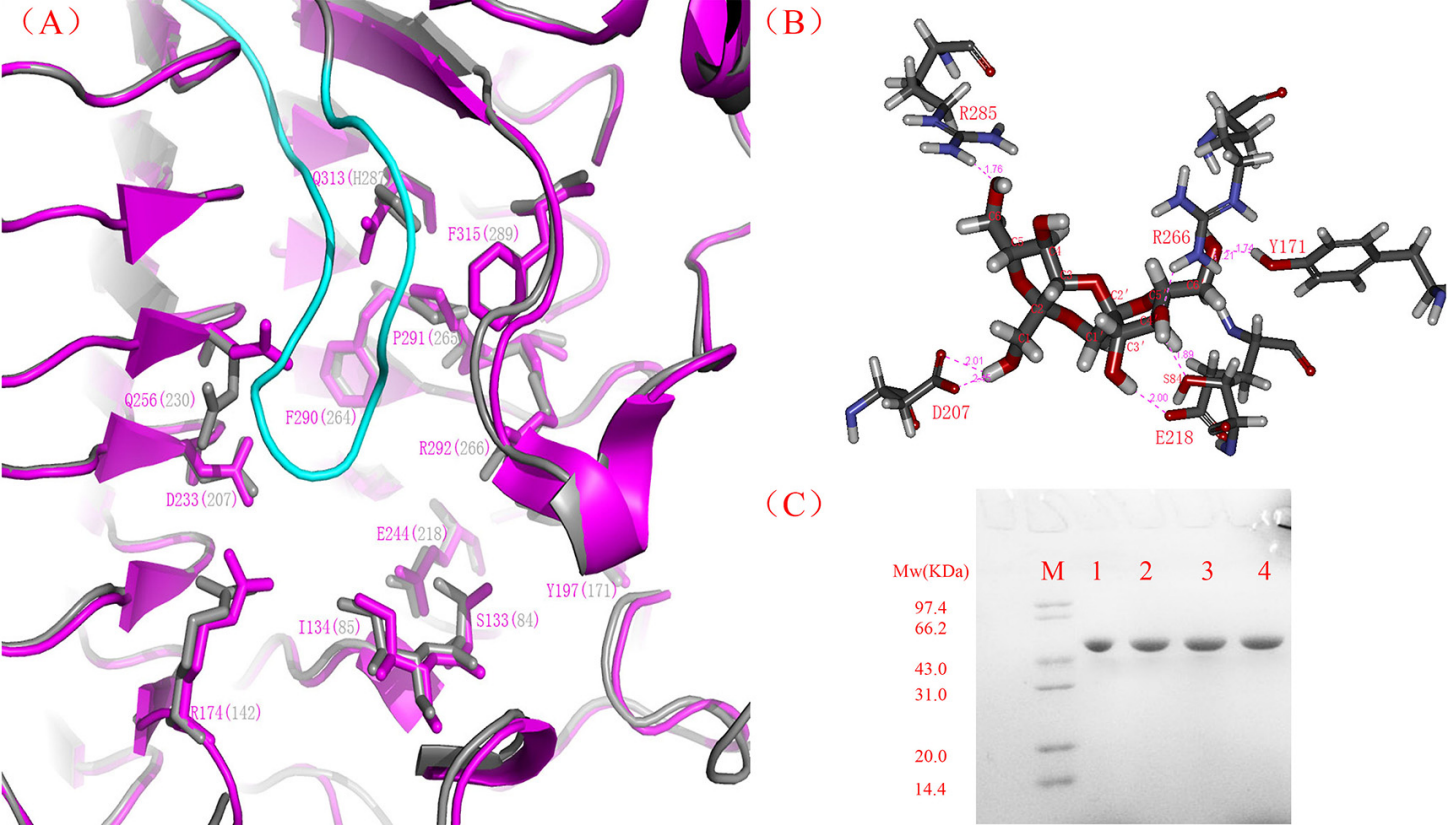
Putative active site of *Aa*DFA IIIase and molecular docking of DFA III into this putative catalytic pocket. (A) The putative reactive pocket and active site residues of *Aa*DFA IIIase based on superimposition of *Aa*DFA IIIase model (gray) and crystal structure of *Bacillus* sp. IFTase (pink). All of active residues of IFTase were showed as pink and putative active residues of *Aa*DFA IIIase in parenthesis as gray. (B) Molecular docking of DFA III into the putative active pocket of *Aa*DFA IIIase model. DFA III was bound into the putative active site through hydrogen bonds (green dotted lines) with R285 (1.76 Å), D207 (2.01 Å), E218 (2.00 Å), S84 (1.89 Å), R266 (2.1 Å), and Y171 (1.74 Å). (C) SDS-PAGE analysis of the mutants. M represents standard protein markers and Arabic numbers 1–4 represented D207A, D207N, E218A, and E218N mutants, respectively.

The overall structures of *Aa*DFA IIIase and its mutants were right-handed parallel *β*-helix structures, which were the same as that of the IFTase reported by Jung [42]. Fig 5B showed that the monomer of *Aa*DFA IIIase model superimposed with the majority of regions of IFTase monomer except two long loop regions. It indicated that the trimerization was probably also a prerequisite for catalytic activity of *Aa*DFA IIIase and that the active site might be located at the monomer-monomer interface as IFTase. Based on this speculation, the constructed model of *Aa*DFA IIIase was superimposed onto the template crystal structure (shown in Fig 6A), and the result showed that, except H287, all the residues of *Aa*DFA IIIase corresponding to the active positions in *Bs*IFTase were completely the same as those of *Bs*IFTase, indicating that these residues in *Aa*DFA IIIase probably form an active pocket that recognizes and catalyzes DFA III.

### Molecular docking and site-directed mutagenesis

Molecular docking of DFA III into the putative active pocket of *Aa*DFA IIIase was performed by AutoDock4.2. The best ligand docking conformation was selected based on the docking energy and root mean square deviation (RMSD) criterion. As shown in Fig 6B, several hydrogen bonds were generated between DFA III and residues of the side-chains of the *Aa*DFA IIIase model. Jung et al. reported that E244 in *Bs*IFTase acts as a general base to activate the acceptor and that D233 orients the donor molecule into an optional configuration for catalysis, suggesting that D233 and E244 play critical roles in catalyzing the biological reaction [42]. Shown in Fig 6B, the two residues D207 and E218, corresponding to the D233 and E244 in *Bs*IFTase, also directly interacts with the substrate DFA III. To determine the functional roles of these two residues, site-mutagenesis was performed, and the mutant enzymes were expressed and purified as the wild-type *Aa*DFA IIIase described above. Each of the purified proteins appeared as a single major band on SDS-PAGE (Fig 6C). The enzymatic activities of various mutants, including D207A, D207N, E218A, and E218N, were measured; however, all of them were completely inactive. Therefore, it was suggested that the residues at position of 207 and 218 were indispensable for the catalytic activity of *Aa*DFA IIIase.

**Fig 7:**
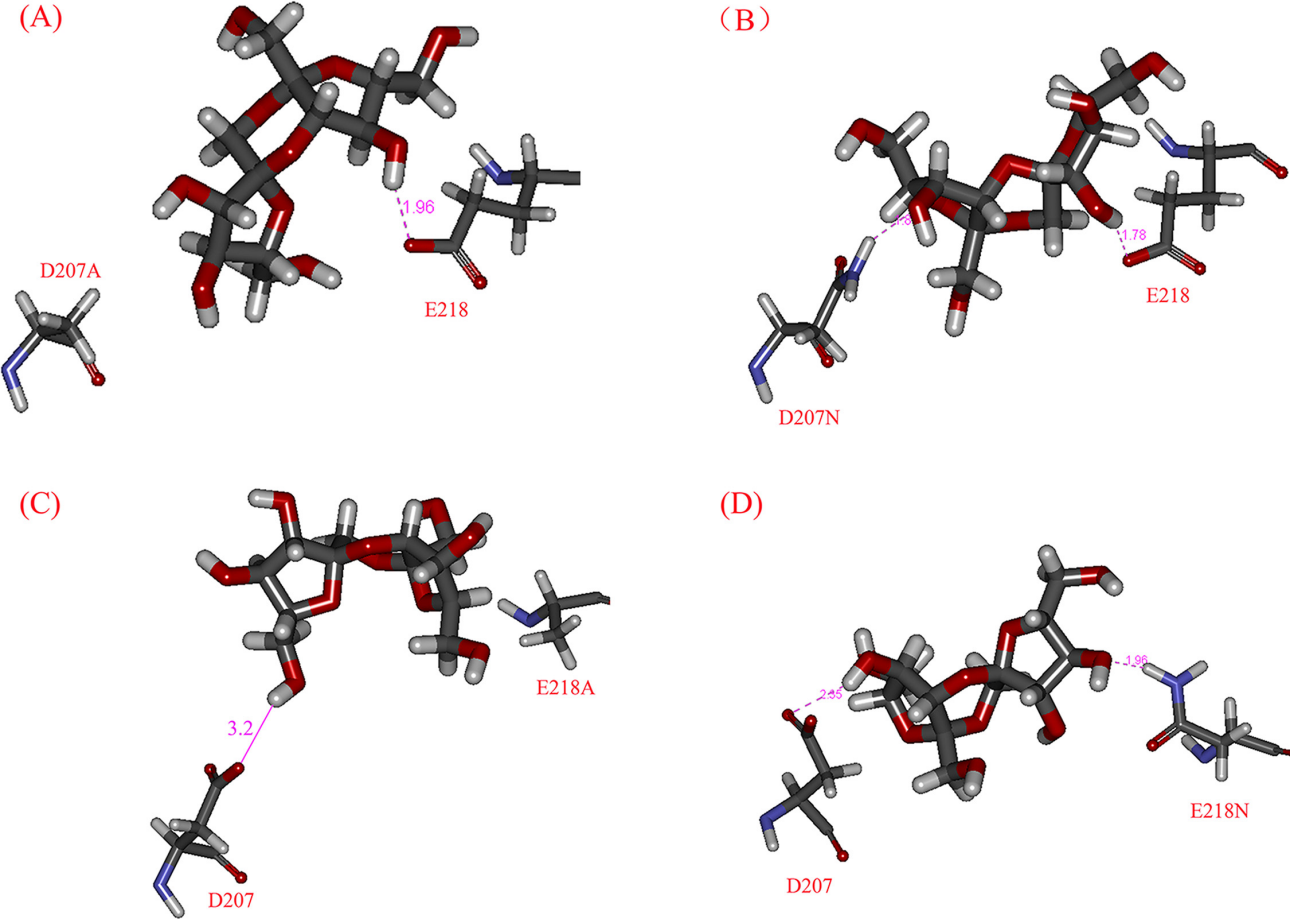
Molecular docking of DFA III to the putative active pocket of *Aa*DFA IIIase mutants. A, B, C, and D represented the molecular docking of D207A, D207N, E218A, and E218N mutants, respectively. The dotted and solid pink lines represented strong and relatively weak hydrogen bonds, respectively. The distances of H bonds were labeled with pink Arabic numbers.

The molecular docking analyses of mutants and DFA III were implemented as the wild-type *Aa*DFA IIIase. For D207 mutants (shown in Fig 7A and 7B), E218 still interacted with DFA III molecule, and no hydrogen bond was generated between D207A and DFA III, while the amino-group of D207N hydrogen bonded with DFA III. For E218 mutants (Fig 7C and 7D), the alanine residue in the mutant E218A also had no hydrogen bond with DFA III, whereas the amino group of E218N hydrogen bonded with DFA III as D207N. Moreover, D207 formed a weak hydrogen bond with DFA III at a distance of 3.2 Å (Fig 7C). The negatively charged residues such as aspartate and glutamate usually act as general bases or nucleophiles. The mutagenesis of these residues probably changes the charge state and inactivates the enzyme, which have been confirmed by many enzymes such as IFTase [42], levan fructotransferase [44] and other glycosyltransferases [45, 46].

Furthermore, multiple sequence alignment of the DFA IIIases and the reported IFTases (DFA I-forming and DFA III-forming) were carried out, as shown in S5 Fig. It is interesting that all residues responding to D207 and E218 of *Aa*DFA IIIase are completely conserved in all sequences. Eleven non-catalytic critical residues of *Bs*IFTase, including S133, I134, R174, Y197, F290, P291, R292, Q256, Q313, F315, and F346, were also highly conserved in these enzymes. It suggests that all of the DFA IIIases and IFTases probably share very similar active pocket and catalytic residues. To evaluate this hypothesis, the comparison of these three-dimensional structures should be performed. However, only the crystallographic structure of *Bs*IFTase has been reported. Hence, the three-dimensional structures of IFTase (DAF I-forming) and DFA IIIase should be further investigated.

In conclusion, a novel DFA IIIase-encoding gene (GenBank accessioin No. KR534324) from *A*. *aurescens* SK 8.001 was cloned and expressed in *E*. *coli*, and the purified DFA IIIase was identified. It showed very high amino acid identity with DFA IIIase from *Arthrobacter* sp. H65-7, but much less identity with all identified IFTases. The enzyme showed the maximum activity at pH 5.5 and 55°C. The specific activity, *K*_m_, and *V*_max_ for DFA III were 232 U/mg, 30.7 ± 4.3 mM and 1.2 ± 0.1 mM min^-1^, respectively. Homology modeling, molecular docking, and site-directed mutagenesis revealed that the residues of D207 and E218 were the potential catalytic site residues for DFA III binding and hydrolysis.

## Supporting Information

S1 Fig The nucleotide sequence of the DFA IIIase from *A*. *aurescens* SK 8.001. This DNA sequence analysis reveals an open reading frame of 1,413 bp, encoding a polypeptide of 470 amino acid residues with a calculated molecular mass of 49,879 Da and isoelectric point of pH 4.56.
(JPG)

S2 Fig The LC-ESI-MS spectrum of the purified product from the reaction mixture. The mass spectrometry system was operated in the negative ion mode with a negative electrospray ionization source (ESI^-^), and the cone voltage, capillary voltage, source temperature, and mass range were 30 V, 3.0 kV, 100°C, and 50–1000 m/z, respectively.
(JPG)

S3 Fig Nonlinear regression plots for the analysis of the kinetic parameters of *Aa*DFA IIIase. Assays were performed in standard conditions using various concentrations of DFA III.
(JPG)

S4 Fig Ramachandran plot of the *Aa*DFA IIIase model analyzed by WinCoot. The analytic result showed that 90.79% of amino acid residues are located within the preferred regions, with 4.72% residues in allowed regions, while 4.49% of residues in the outlier regions of Ramachandran plot.
(JPG)

S5 Fig Multiple sequence alignment of DFA IIIases and IFTases. The Arabic numbers before each sequence represent different enzymes. Nos. 1–2 represented DFA IIIases from *A*. *aurescens* SK 8.001 (GenBank accession No.: KR534324) and *Arthrobacte*r sp. H65-7 (BAD06469), respectively; No. 3 represented IFTase (DFA I-forming) from *A*. *globiformis* S14-3 IFTase (BAA07533); Nos. 4–11 represented IFTases (DFA III-forming) from *Arthrobacter* sp. 161MFSha2.1 (WP_018778058), *A*. *aurescens* SK 8.001 (ADJ19283.1), *Arthrobacter* sp. L68-1 (BAO57215), A. *globiformis* C11-1 (BAB20662), *Bacillus* sp. snu-7 (AAZ66341), *Arthrobacter* sp. A-6 (AF124980_1), *Arthrobacter* sp. H65-7 (BAA18967) and *Nonomuraea* sp. ID06-A0189 (BAN62836), respectively. Red stars represent the residues responding to D207 and E218 of AaDFA IIIase in different enzymes. The residues responding to active site of *Bacillus* sp. snu-7 IFTase (crystallographic structure PDB ID: 2INU) were labeled with ■ above the sequences. Cyan and pink backgrounds represented the identity of amino acid sequences of more than 50% and 75%, respectively. Black background indicated all the completely conserved residues and they were labeled under the sequences with lowercase letters. The alignment was generated with DNAman (LynnonBiosoft, USA).
(JPG)
