# Supplementary figures and images for: Identification of a Novel Di-D-Fructofuranose 1,2’:2,3’ Dianhydride (DFA III) Hydrolysis Enzyme from Arthrobacter aurescens SK8.001
Source: PLoS One. 2015 Nov 10;10(11):e0142640. doi: 10.1371/journal.pone.0142640 (PMC4640833; doi:10.1371/journal.pone.0142640)

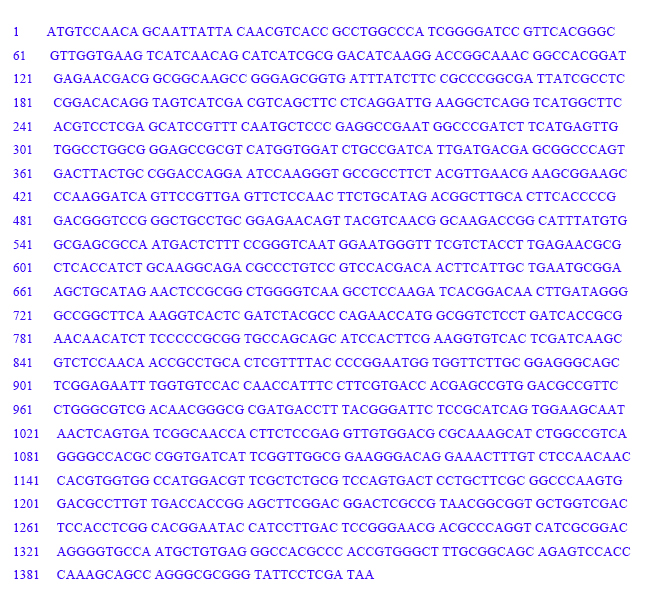

Supplement: S1 Fig — This DNA sequence analysis reveals an open reading frame of 1,413 bp, encoding a polypeptide of 470 amino acid residues with a calculated molecular mass of 49,879 Da and isoelectric point of pH 4.56. (JPG) [file pone.0142640.s001.jpg]

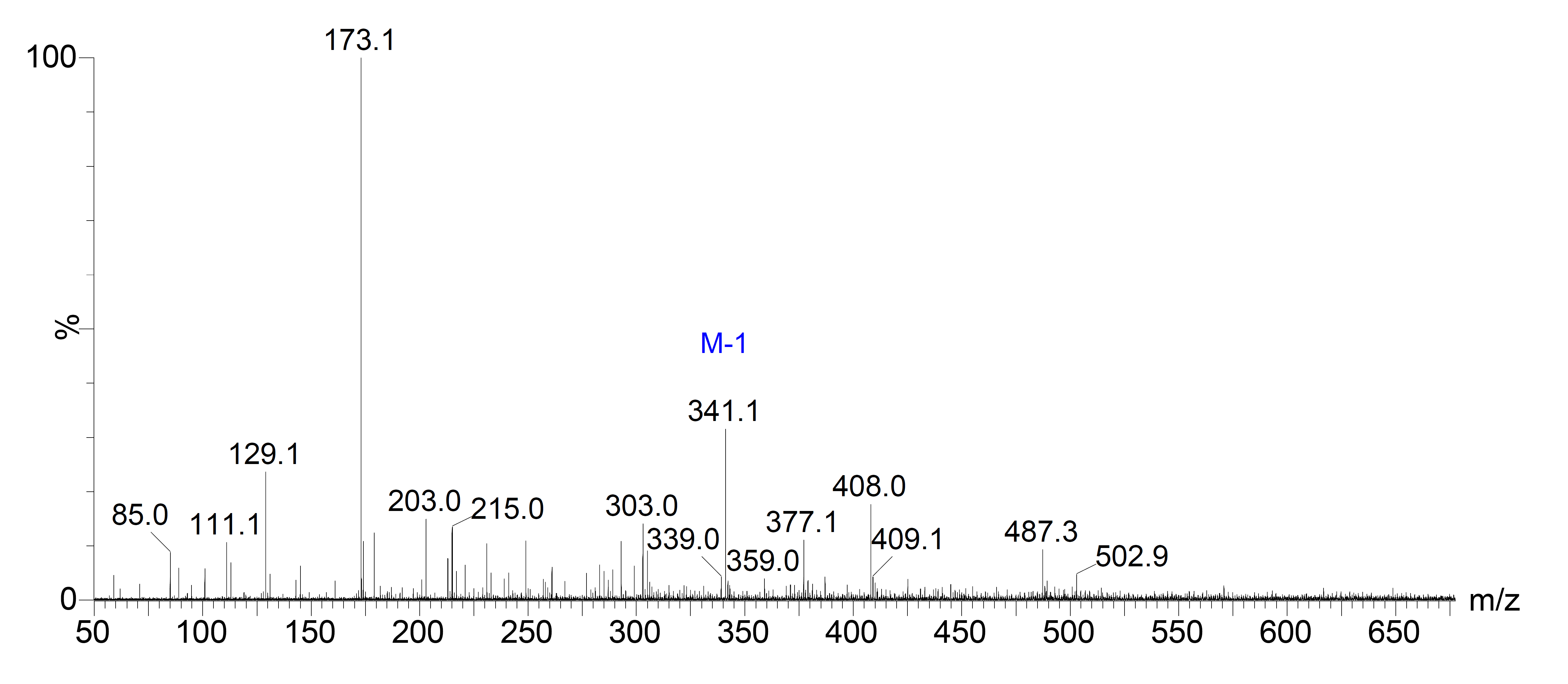

Supplement: S2 Fig — The mass spectrometry system was operated in the negative ion mode with a negative electrospray ionization source (ESI-), and the cone voltage, capillary voltage, source temperature, and mass range were 30 V, 3.0 kV, 100°C, and 50–1000 m/z, respectively. (JPG) [file pone.0142640.s002.jpg]

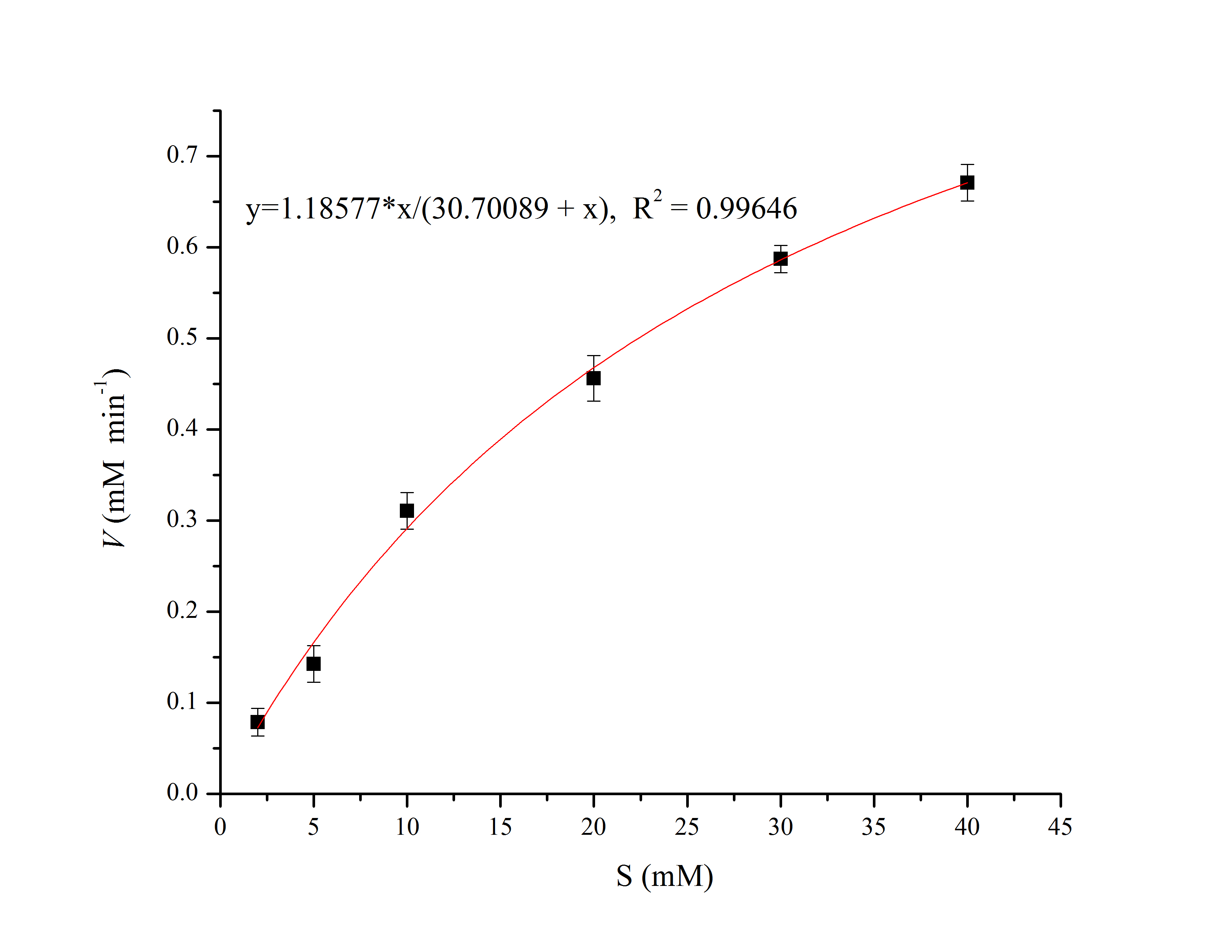

Supplement: S3 Fig — Assays were performed in standard conditions using various concentrations of DFA III. (JPG) [file pone.0142640.s003.jpg]

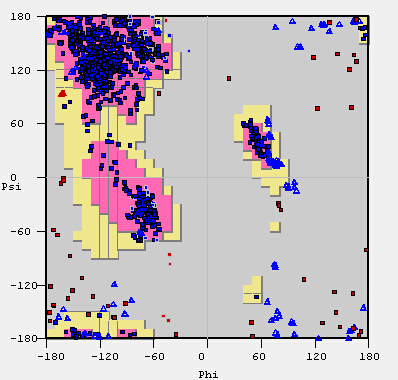

Supplement: S4 Fig — The analytic result showed that 90.79% of amino acid residues are located within the preferred regions, with 4.72% residues in allowed regions, while 4.49% of residues in the outlier regions of Ramachandran plot. (JPG) [file pone.0142640.s004.jpg]

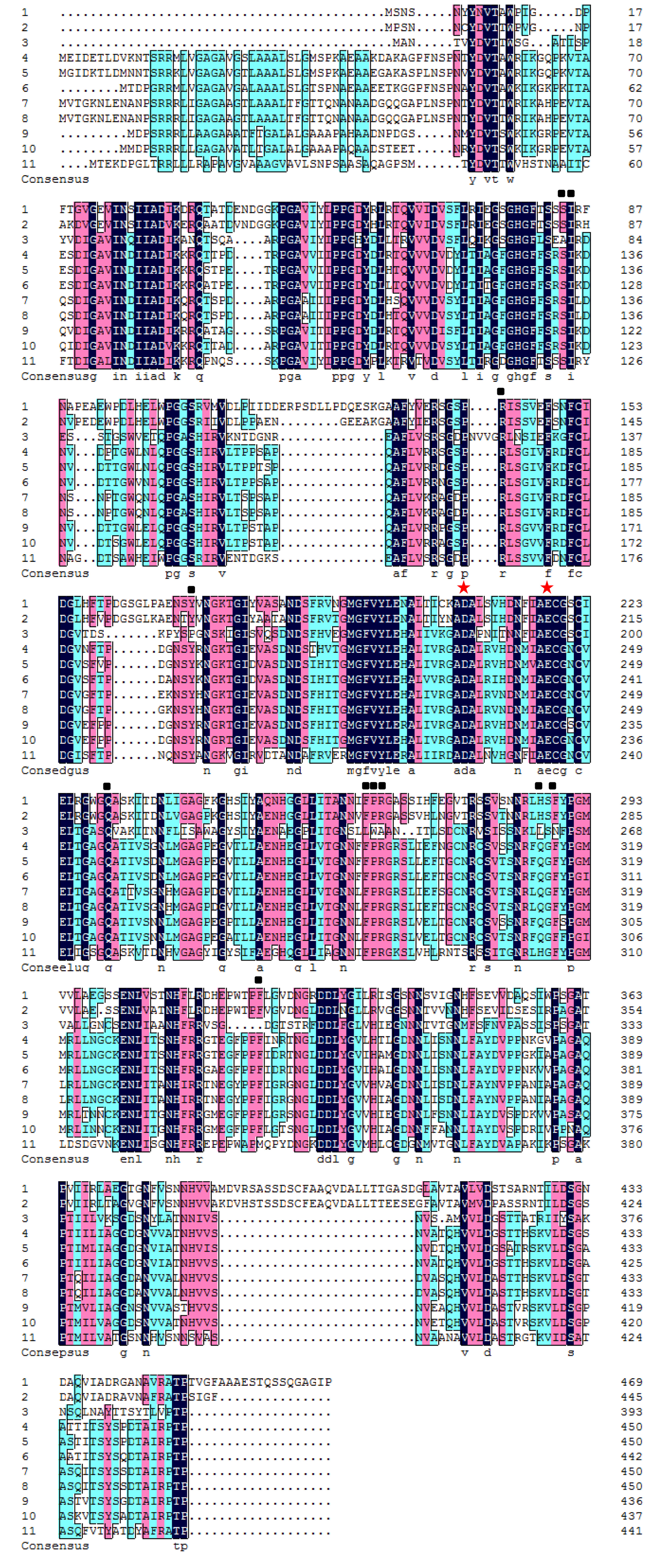

Supplement: S5 Fig — The Arabic numbers before each sequence represent different enzymes. Nos. 1–2 represented DFA IIIases from A. aurescens SK 8.001 (GenBank accession No.: KR534324) and Arthrobacter sp. H65-7 (BAD06469), respectively; No. 3 represented IFTase (DFA I-forming) from A. globiformis S14-3 IFTase (BAA07533); Nos. 4–11 represented IFTases (DFA III-forming) from Arthrobacter sp. 161MFSha2.1 (WP_018778058), A. aurescens SK 8.001 (ADJ19283.1), Arthrobacter sp. L68-1 (BAO57215), A. globiformis C11-1 (BAB20662), Bacillus sp. snu-7 (AAZ66341), Arthrobacter sp. A-6 (AF124980_1), Arthrobacter sp. H65-7 (BAA18967) and Nonomuraea sp. ID06-A0189 (BAN62836), respectively. Red stars represent the residues responding to D207 and E218 of AaDFA IIIase in different enzymes. The residues responding to active site of Bacillus sp. snu-7 IFTase (crystallographic structure PDB ID: 2INU) were labeled with ■ above the sequences. Cyan and pink backgrounds represented the identity of amino acid sequences of more than 50% and 75%, respectively. Black background indicated all the completely conserved residues and they were labeled under the sequences with lowercase letters. The alignment was generated with DNAman (LynnonBiosoft, USA). (JPG) [file pone.0142640.s005.jpg]
